# The response of apoptotic and proteolytic systems to repeated heat stress in atrophied rat skeletal muscle

**DOI:** 10.14814/phy2.12597

**Published:** 2015-10-27

**Authors:** Toshinori Yoshihara, Takao Sugiura, Yuki Yamamoto, Tsubasa Shibaguchi, Ryo Kakigi, Hisashi Naito

**Affiliations:** 1 Graduate School of Health and Sports Science, Juntendo UniversityInzai, Chiba, Japan; 2 Faculty of Education, Yamaguchi UniversityYoshida, Yamaguchi, Japan; 3 Faculty of Health and Sport Sciences, University of TsukubaTsukuba, Ibaraki, Japan; 4 Graduate School of Frontier Biosciences, Osaka UniversityToyonaka, Osaka, Japan; 5 Faculty of Medicine, Juntendo UniversityBunkyo-ku, Tokyo, Japan

**Keywords:** Apoptosis, disuse muscle atrophy, heat shock protein, protein degradation

## Abstract

We examined the effect of repeated heat stress on muscle atrophy, and apoptotic and proteolytic regulation in unloaded rat slow- and fast-type skeletal muscles. Forty male Wistar rats (11 week-old) were divided into control (CT), hindlimb unweighting (HU), intermittent weight-bearing during HU (HU + IWB), and intermittent weight-bearing with heat stress during HU (41–41.5°C for 30 min; HU + IWB + HS) groups. The HU + IWB + HS and HU + IWB groups were released from unloading for 1 h every second day, during which the HU + IWB + HS group underwent the heating. Our results revealed that repeated bouts of heat stress resulted in protection against disuse muscle atrophy in both soleus and plantaris muscles. This heat stress–induced protection against disuse-induced muscular atrophy may be partially due to reduced apoptotic activation in both muscles, and decreased ubiquitination in only the soleus muscle. We concluded that repeated heat stress attenuated skeletal muscle atrophy via suppressing apoptosis but the response to proteolytic systems depend on the muscle phenotype.

## Introduction

Skeletal muscle mass is balanced between protein synthesis and degradation. Protein synthesis and breakdown are regulated through multiple signaling pathways; decreased protein synthesis and/or increased protein degradation contribute to loss of muscle protein (Thomason and Booth 1990; Booth and Criswell 1997). Although the protein synthetic rate declines rapidly following hindlimb unloading, disuse-induced muscle atrophy is mainly due to an increase in the protein degradation rate in rat skeletal muscle (Booth and Criswell 1997; Powers et al. 2005, 2007). In general, three proteolytic pathways are involved in muscle protein degradation, namely the lysosomal (e.g., cathepsin L and H), Ca^2+^-dependent (e.g., calpain), and ubiquitin–proteasome systems (Taillandier et al. 1996; Powers et al. 2005, 2007). Furthermore, recent evidence has revealed that the apoptotic system may also contribute to select forms of disuse muscle atrophy (Du et al. 2004; McClung et al. 2007; Powers et al. 2007).

Numerous studies have indicated that apoptosis and proteolysis-related proteins and genes increase during the early stages of disuse-induced skeletal muscle atrophy. Dupont-Versteegden et al. (Dupont-Versteegden et al. 2006) demonstrated that the increases in apoptotic markers and muscle atrophy-specific ubiquitin E3 ligase (MAFbx) occurred as early as 12 h after suspension. Furthermore, calpain activity, which is induced by increased cytosolic-free Ca^2+^ concentration, increased in rat soleus muscle following 12 h of hindlimb unloading and was maintained for 9 days (Enns and Belcastro 2006). These results suggest that suppressing these increases in the apoptotic and proteolytic systems may be important to effectively attenuating disuse-induced muscle atrophy.

Heat stress has attracted attention as an effective treatment against disuse-induced muscle atrophy. Naito et al. (Naito et al. 2000) demonstrated that one bout of heat stress prior to hindlimb unweighting attenuates rat soleus muscle atrophy. Moreover, Selsby et al. (Selsby and Dodd 2005) reported that intermittent (repeated) heat stress during hindlimb immobilization reduced oxidative stress in atrophied soleus muscle via increased expression of heat shock proteins (HSPs) (Selsby and Dodd 2005). Given that repeated heat stress reduces the disuse-related increase in oxidative stress, repeated heat-stress treatment may protect against the upregulation of apoptosis and proteases that occurs during the early stage of disuse. However, there is no evidence that repeated heat stress attenuates the acceleration of apoptotic and proteolytic systems in atrophied rat skeletal muscle.

Therefore, the aim of this study was to examine the effects of repeated heat stress on muscle atrophy and in terms of apoptosis and proteolysis in unloaded rat skeletal muscle induced by hindlimb unloading. Additionally, we examined whether two different muscles, a slow-type muscle, which atrophies quickly, and a fast-type muscle, which is less sensitive to disuse, show the same pattern of effects following repeated heat stress.

## Methods

### Experimental animals and heat treatment

This study was approved by the Yamaguchi University Animal Care Committee and followed the guiding principles for the care and use of animals set forth by the Physiological Society of Japan. Forty male Wistar rats (age, 11 weeks; weight, 396.5 ± 4.25 g) were used. The rats were housed in a climate-controlled room (24.0 ± 1°C, 50–60% relative humidity, 12:12-h light–dark photoperiod) and were given standard rat chow and water ad libitum. The rats were divided randomly into four groups: control (CT, *n *=* *10), hindlimb unweighting (HU, *n *=* *10), intermittent weight-bearing during HU (HU + IWB, *n *=* *10), and intermittent weight-bearing with heat stress during HU (HU + IWB + HS, *n *=* *10) groups. The HU, HU + IWB, and HU + IWB + HS groups were unloaded for 7 days, as described previously (Sugiura et al. 2005). Briefly, a tail cast was applied to each rat, leaving the distal one-third of the tail free to allow for proper thermoregulation. The tail cast was attached to a hook on the ceiling of the cage, and the height of the hook was adjusted at an inclination of approximately 35° in a head-down orientation. The rat was free to move around the cage on its front feet. Rats were checked daily for signs of tail lesions or discoloration. During hindlimb unweighting, the HU + IWB + HS group was released from unweighting for 1 h every second day for heating; the HU + IWB group experienced similar unweighting to that of the HU + IWB + HS group. One day before and during weight-bearing, the HU + IWB + HS group was placed in a heat chamber (IS-2400, ADVANTEC, Tokyo, Japan) for 30 min (41–41.5°C). Mean rectal temperature of the heat-treated rats was 41.2 ± 0.2°C at the end of the heat treatment. After the experimental period, all rats were anesthetized with ether; the soleus and plantaris muscles were removed from both legs, frozen rapidly in liquid nitrogen, and stored at −80°C until analysis.

### Protein isolation and Western blot analysis

#### Muscle preparation

Frozen soleus and plantaris muscle samples (∼90 mg) were minced and homogenized in 10 volumes of ice-cold homogenization buffer (20 mmol/L Tris-HCl, pH 7.4, 25 mmol/L KCl, 5 mmol/L EDTA, 5 mmol/L EGTA, and 1 mmol/L dithiothreitol) containing a protease inhibitor cocktail (Nacalai Tesque, Inc., Kyoto, Japan). The homogenates were centrifuged at 1000 *g* for 10 min at 4°C, and the supernatant was collected as the soluble fraction. The protein concentration in the supernatant was determined using a Protein Assay kit (Bio-Rad, Hercules, CA). The insoluble pellets corresponding to the particulate fraction were suspended in lysis buffer using the method of Solaro et al. (Solaro et al. 1971). The protein concentration of the solubilized particulate fraction was determined using the Biuret method.

#### Sodium dodecyl sulfate–polyacrylamide gel electrophoresis (SDS-PAGE), Western blot, and immunodetection

Proteins were loaded onto 7.5–10% SDS-PAGE gels and transferred onto a polyvinylidene difluoride membrane (Amersham, Buckinghamshire, UK). After transferring the proteins, the membranes were blocked with 5% nonfat dry milk and incubated with primary antibodies overnight at 4°C. The primary antibodies used for the assay included: HSP70 (1:1000; Stressgen, Victoria, BC, CAN), desmin (1:500; Novocastra, Newcastle upon Tyne, UK), cathepsin L (1:1000; BioVision, Milpitas, CA), calpain 1 (1:1000; Cell Signaling Technology, Beverly, MA), calpain 2 (1:1000; Sigma, St. Louis, MO), and multi-ubiquitin (1:500; Stressgen). Then, the membranes were incubated with anti-rabbit or anti-mouse horseradish peroxidase-conjugated secondary antibodies (1:20,000; Sigma) for 2 h at room temperature. Each band was revealed using the ECL Plus reagents (GE Healthcare, Piscataway, NJ), and the signal was recorded by an ATTO Light Capture system (ATTO, Tokyo, Japan). Band intensities were quantified by CS ANALYZER 3.0 software (ATTO).

To analyze HSP72 expression levels, the membranes were incubated with anti-HSP70 alkaline phosphatase conjugate in T-TBS with 5% nonfat dry milk for 1 h at room temperature to analyze HSP72 expression. The membranes were reacted with alkaline phosphatase substrate (Bio-Rad) at room temperature, and the bands were quantified using computerized densitometry (Scion Image; National Institutes of Health, Bethesda, MD). Expression of these proteins is expressed as a percentage of the CT-Sed value.

### Histology and immunohistochemistry

#### Cross-sectional area determination

Cross sections of the soleus and plantaris muscles (*n *=* *6 per group) were cut on a cryostat (8 *μ*m), air-dried, and stored at −20°C until further analysis. The sections were rehydrated in PBS with 0.1% Tween 20, and standard hematoxylin and eosin staining was performed to measure total myofiber Cross-sectional area (CSA). The muscle sections were viewed and captured as digital images using a fluorescence microscope (ECLIPSE E400, Nikon, Tokyo, Japan) equipped with a CCD camera (C120-PC, Leica, Wetzlar, Germany). CSA was determined on 400–500 fibers from five different areas of the mid-belly region of the soleus and plantaris muscles using the Scion Image software package (NIH, Bethesda, MD), and mean myofiber CSA was calculated.

#### Determination of nuclear apoptosis

Nuclei exhibiting apoptotic changes were identified by terminal deoxynucleotidyl transferase dUTP nick end labeling (TUNEL) assay using the in situ cell death detection kit, TMRred (Roche Applied Science, Indianapolis, IN) according to the manufacturer’s recommendations. Briefly, the sections were rehydrated in PBS with 0.1% Tween 20 and then fixed in 4% paraformaldehyde at room temperature for 20 min. After several washes with T-PBS, the sections were fixed in 0.1% (w/v) sodium citrate (0.1% C_5_H_3_O_7_Na_3_·2H_2_O and 0.1% Triton X-100) at room temperature for 2 min. After washing, the sections were immunoreacted with anti-laminin antibody (1:500, Dako Japan, Tokyo, Japan) diluted in 1% (w/v) blocking reagent (Roche) in maleic acid buffer (0.1 mol/L maleic acid and 0.15 mol/L NaCl, pH 7.5) at room temperature for 1 h. After several washes in T-PBS, a fluorescein isothiocyanate-conjugated goat anti-rabbit IgG secondary antibody (1:250, Sigma) in Blocking buffer (Roche) was applied for 1 h at room temperature. After several washes in T-PBS, the TUNEL reaction mix diluted in TUNEL Dilution Buffer (Roche) was applied and incubated at 37°C for 60 min in the dark. After several washes in T-PBS, the sections were cover slipped using Vectashield mounting medium with 4′, 6-diamidino-2- phenylindole (Vector Labs, Burlingame, CA).

### Statistical analysis

Values are presented as means ± standard errors. Differences were detected using one-way analysis of variance (ANOVA). When the ANOVA was significant, group differences were identified using the Tukey–Kramer post hoc test. *P*-values <0.05 were considered significant. All analyses were performed using the Prism software package (ver. 6.0; GraphPad Software Inc., San Diego, CA).

## Results

### Body weight, muscle weight, and myofiber cross-sectional area

Body weights in the HU + IWB and HU + IWB + HS groups were significantly lower than that in the CT group, but no differences were observed among the three hindlimb unweighting groups (Table1). Although 7 days of hindlimb unloading resulted in a significant reduction in soleus (−31%) and plantaris (−19%) muscle weights, soleus and plantaris muscle weights in the HU + IWB + HS group were significantly higher than those in the HU group (Table1). Moreover, relative muscle weight of both muscles in the HU + IWB + HS group was significantly greater than that in the HU and HU + IWB groups.

**Table 1:** Body weight, muscle weight and CSA of the soleus and plantaris muscles after 7-days hind limb unloading

	CT	HU	HU + IWB	HU + IWB + HS
Body weight (g)	408.9 ± 6.7	384.0 ± 9.2	379.3 ± 6.0*	356.3 ± 7.7*
Muscle weight (mg)				
SOL	168.2 ± 6.7	116.3 ± 3.1*	121.1 ± 3.7*	131.1 ± 2.4*†
PLA	435.0 ± 11.1	353.0 ± 9.3*	370.5 ± 7.2*	367.7 ± 10.2*
Relative muscle weight (mg/kg)				
SOL	410.7 ± 12.8	303.8 ± 9.3*	318.9 ± 7.7*	368.7 ± 7.4*†‡
PLA	1063.7 ± 20.3	920.0 ± 16.1*	977.5 ± 17.5*	1032.6 ± 22.0†‡
CSA (*μ*m^2^)				
SOL	3977.3 ± 195.7	2282.2 ± 136.2*	2274.3 ± 184.8*	3338.9 ± 421.7†‡
PLA	3330.1 ± 261.9	2581.1 ± 82.0*	2510.6 ± 92.1*	2839.6 ± 187.9

*n *=* *10 (*n *=* *6 for CSA) per group. CSA, cross-sectional area; CT, control; HU, hindlimb unweighting; HU + IWB, intermittent weight-bearing during HU; HU + IWB + HS, intermittent weight-bearing with heat stress during HU.

* *P* < 0.05 versus CT

† *P* < 0.05 versus HU

‡ *P* < 0.05 versus HU + IWB.

Hindlimb unloading resulted in a significant reduction in soleus (−43%) and plantaris (−23%) myofiber CSA (Table1). Soleus muscle CSA in the HU + IWB + HS group was significantly higher than that in the HU and HU + IWB groups. No significant change was observed in plantaris muscle CSA in the HU + IWB + HS group compared with that in the CT-Sed group.

### HSP72 and desmin protein expression

**Figure 1:**
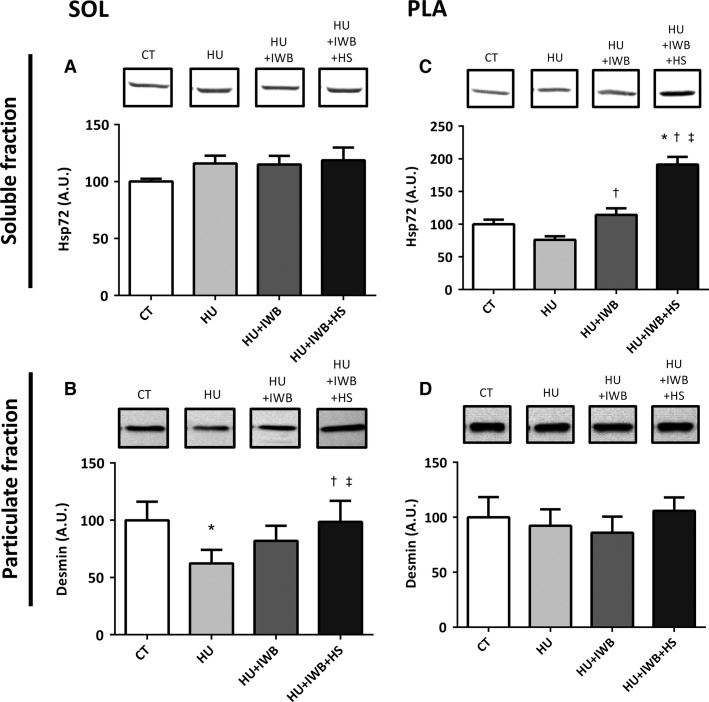
Representative Western blots and Hsp72 in soluble fraction (A and C) and Desmin in particulate fraction (B and D) in the soleus and plantaris muscles after 7 days of hindlimb unloading. The experimental group data are presented as a percentage of the mean CT value, which was set to 100%. *n *=* *10 per group. *P <* *0.05 versus CT; ^†^*P *<* *0.05 versus HU; ^‡^*P *<* *0.05 versus HU + IWB.

No significant difference in soleus muscle HSP72 expression was detected among the experimental groups (Fig.1). In contrast, plantaris muscle HSP72 expression in the HU + IWB and HU + IWB + HS groups increased significantly compared with that in the HU group. Moreover, HSP72 protein expression in the HU + IWB + HS group was significantly higher than that in any other group.

Desmin protein expression in the soleus muscle decreased significantly in the HU group compared with that in the CT group, but desmin protein expression in the HU + IWB + HS group was significantly higher than that in the HU and HU + IWB groups. In contrast, no change was observed in desmin protein expression in the plantaris muscle among the experimental groups.

### TUNEL-positive nuclei

**Figure 2:**
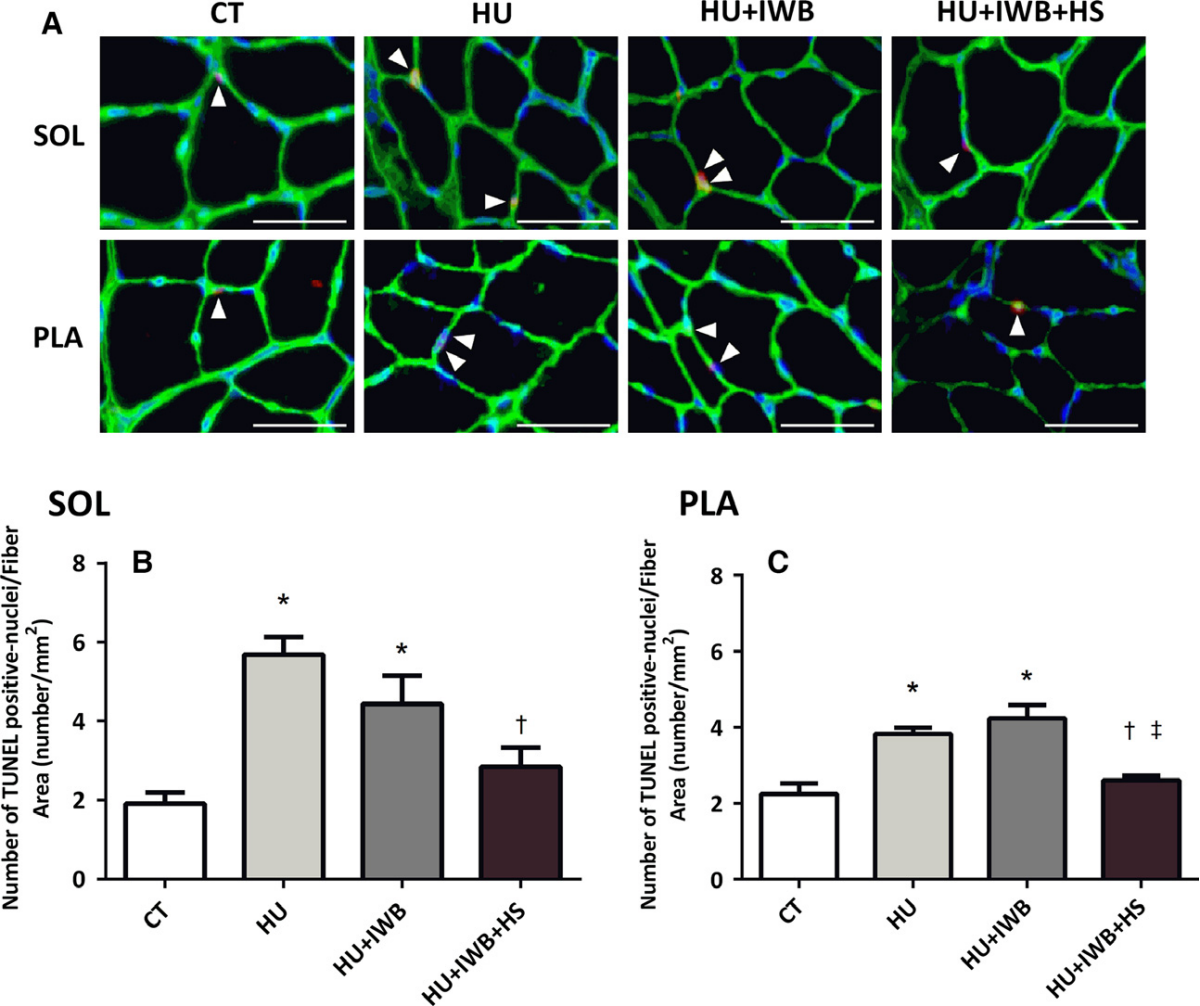
Apoptosis in the soleus and plantaris muscles were determined by the terminal deoxynucleotidyl transferase dUTP nick-end labeling (TUNEL) assay. (A) representative images of sections stained using the TUNEL assay. Soleus and plantaris muscles cross sections were immunoreacted with TUNEL-positive nuclei (red), DAPI (blue), Laminin (green) and a composite is depicted in Figure2A. TUNEL-positive interstitial cells were observed in soleus muscles (arrow). Bar in the picture is 50 *μ*m. TUNEL-positive nuclei after 7 days of hindlimb unloading in the soleus (B) and plantaris (C) muscles. *n *=* *6 per group. *P <* *0.05 versus CT; ^†^*P *<* *0.05 versus HU; ^‡^*P *<* *0.05 versus HU + IWB.

The TUNEL assay was performed to investigate the onset and progression of apoptosis. TUNEL-positive nuclei were located on the laminin basement membrane, overlapping the nucleus, and were counted as apoptotic muscle nuclei. The numbers of TUNEL-positive nuclei per CSA of soleus and plantaris muscle fibers increased significantly in the HU and HU + IWB groups after 7 days of hindlimb unloading (Fig.2B and D). However, the increase was significantly attenuated in plantaris muscle from the HU + IWB + HS group. The number of TUNEL-positive nuclei per CSA of soleus muscle fibers was significantly lower in the HU + IWB + HS group than in the HU group.

### Cathepsin L expression

**Figure 3:**
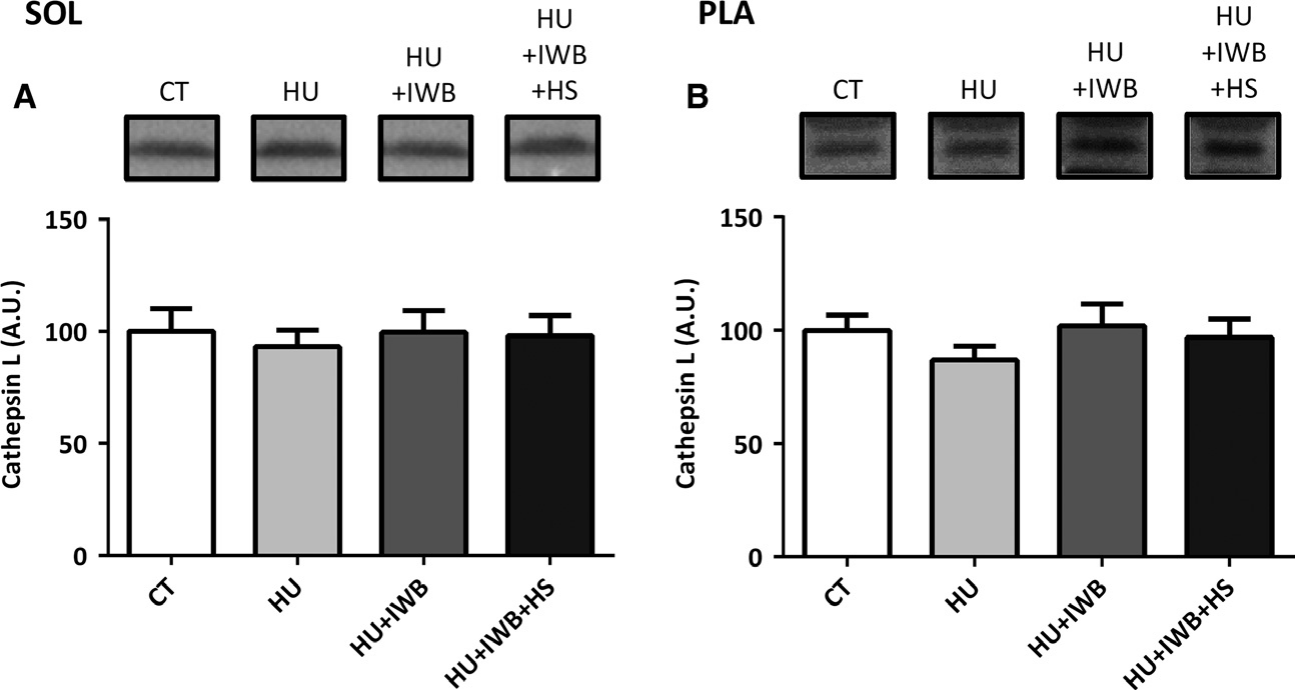
Representative Western blots and soleus (A) and plantaris (B) muscles cathepsin L expression after 7 days of hindlimb unloading. *n *=* *10 per group.

Figure3 shows cathepsin L expression in the soleus (A) and plantaris (B) muscles following the experimental period. No significant differences in cathepsin L expression were observed in either the soleus or plantaris muscle among the experimental groups.

### Calpain 1 and 2 expression and calpain autolysis

**Figure 4:**
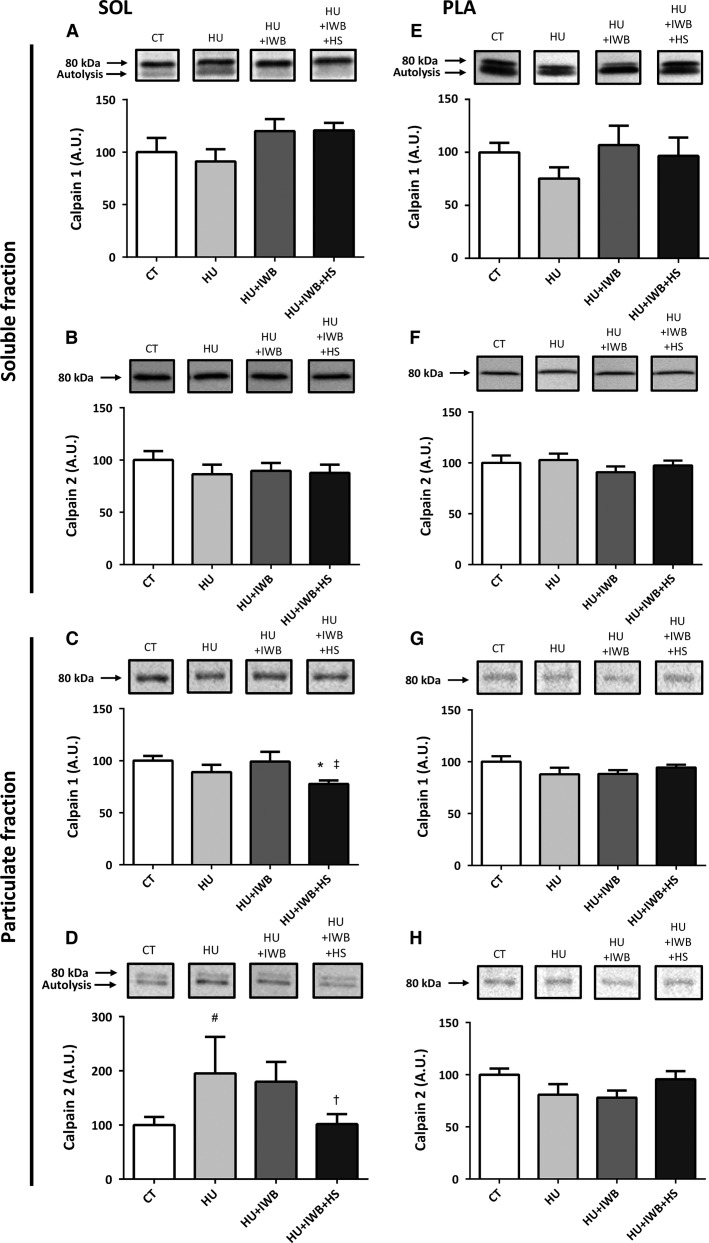
Representative Western blots and total calpain 1 and 2 expression in the soluble and particulate fractions of the soleus (A–D) and plantaris (E–H) muscles after 7 days of hindlimb unloading. Calpain 1 (A and E) and calpain 2 (B and F) in the soluble fractions of the soleus and plantaris muscles, respectively. Calpain 1 (C and G) and calpain 2 (D and H) in the particulate fractions of the soleus and plantaris muscles, respectively. *n *=* *10 per group. *P <* *0.05 versus CT; ^†^*P *<* *0.05 versus HU; ^‡^*P *<* *0.05 versus HU + IWB; ^#^*P *<* *0.10 versus HU.

Total calpain 1 content in the soleus muscle particulate fraction decreased significantly in the HU + IWB + HS group compared with that in the CT and HU groups (Fig.4C), although calpain 1 and 2 content in the soluble fraction remained unchanged (Fig.4A and B). Additionally, calpain 2 expression in the particulate fraction (Fig.4D, *P* = 0.053) tended to increase in the HU group, but no increase was detected in the HU + IWB + HS group. No significant changes in calpain 1 or 2 were observed in the soluble or the particulate fractions of the plantaris muscle (Fig.4E–H) among the experimental groups.

**Figure 5:**
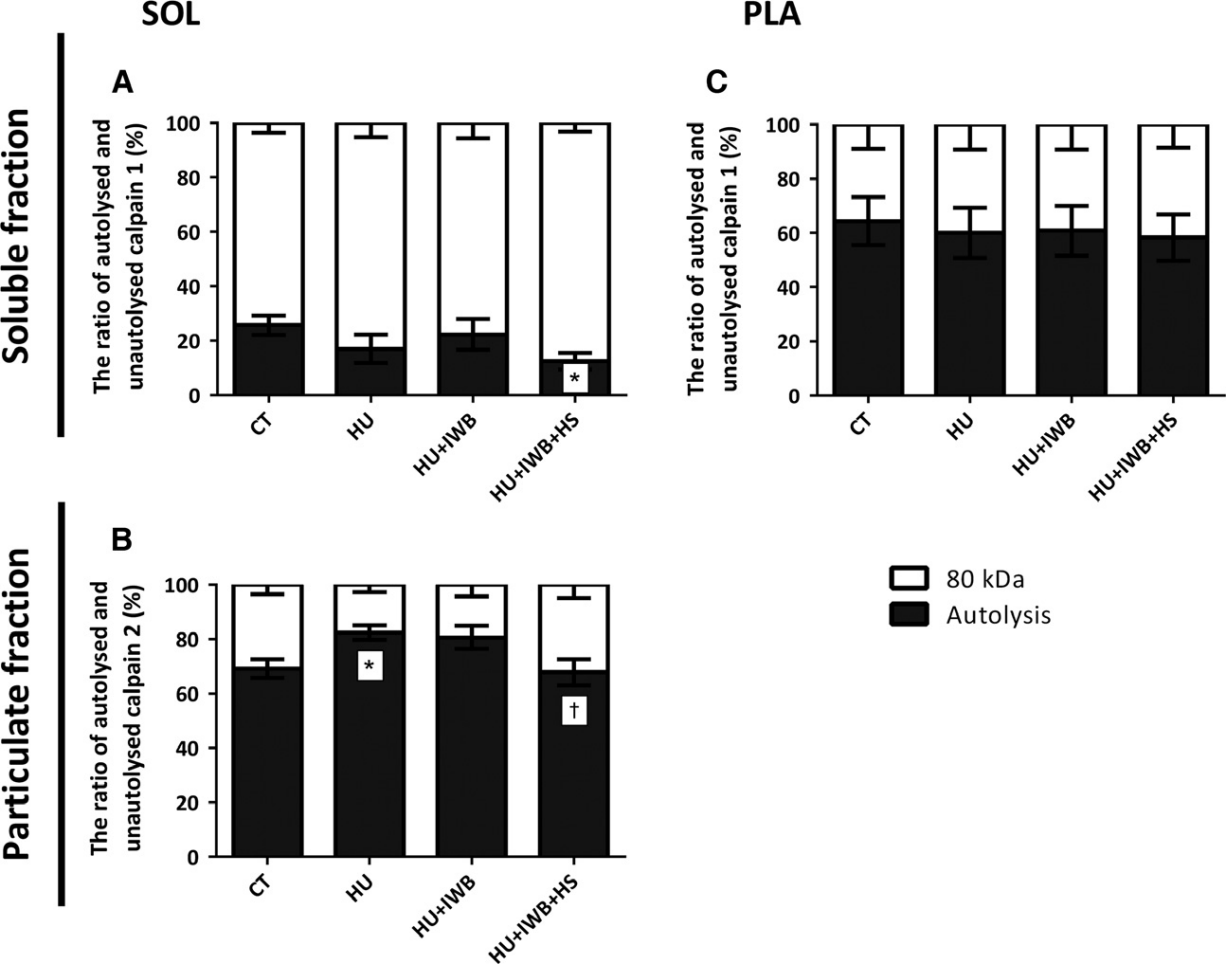
Ratio of autolyzed and unautolyzed (80 kDa) calpain 1 (A and C) in the soluble fraction and calpain 2 (B) in the particulate fraction. Calpain 1 in the soleus muscle soluble fraction (A). Calpain 2 in the soleus muscle particulate fraction (B). Calpain 1 in the plantaris muscle soluble fraction (C). The experimental group data are presented as a percentage of the mean CT value, which was set to 100%. *n* = 10 per group.*P < 0.05 versus CT; ^†^*P* < 0.05 versus HU.

Calpain contains an 80-kDa catalytic subunit and a 28-kDa regulatory subunit, and autolysis of the 80-kDa subunit increases calpain sensitivity to Ca^2+^ in vitro; therefore, calpain autolysis was used as a marker of activation. Autolyzed calpain in the soleus muscle soluble fraction in the HU + IWB + HS group was significantly lower than that in the CT group (Fig.5). Moreover, the autolyzed-form of calpain 2 increased significantly in the particulate fraction of the HU + IWB group, but not in the HU + IWB + HS group, compared with that in the HU group. In the particulate fraction from the plantaris muscle, no calpain 2 autolysis was observed in either group.

### Ubiquitinated protein expression

**Figure 6:**
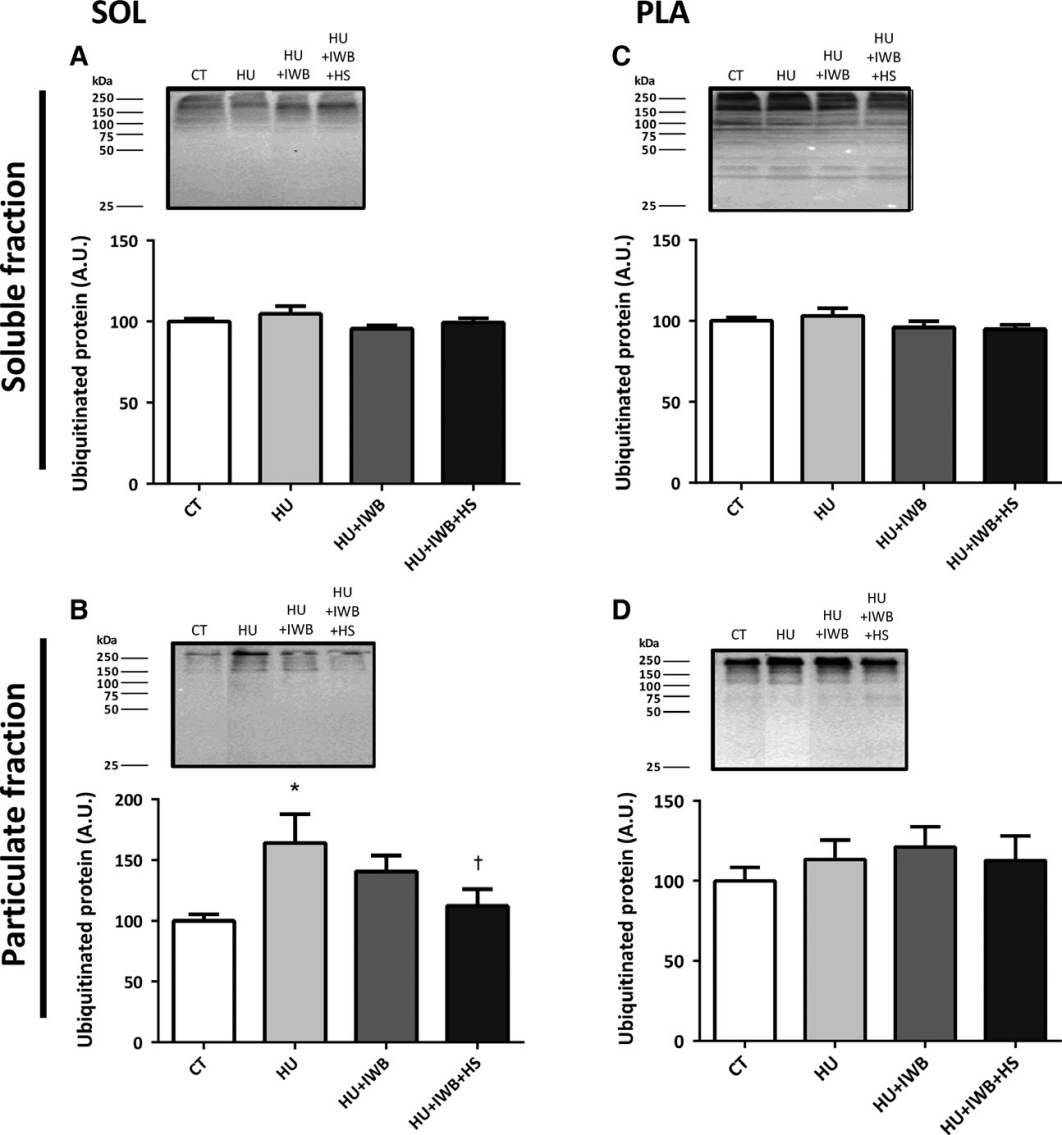
Representative Western blots and ubiquitinated protein expression in the soleus and plantaris muscles soluble (A and C) and particulate (B and D) fractions after 7 days of hindlimb unloading. *n *=* *10 per group. *P <* *0.05 versus CT; ^†^*P *<* *0.05 versus HU.

No significant differences in ubiquitinated protein expression were observed in the soluble fractions of the soleus or plantaris muscles (Fig.6A and C). Ubiquitinated protein increased significantly in the soleus muscle particulate fraction from the HU group after 7 days of hindlimb unloading. However, the increase in ubiquitinated protein was significantly attenuated in the HU + IWB + HS group (Fig.6B). No significant difference was found in the plantaris muscle particulate fraction (Fig.6D).

## Discussion

### The effect of intermittent weight-bearing on the degree of muscle atrophy

One study reported that ubiquitin ligase mRNA expression (e.g., MAFbx and MuRF1) is suppressed by 4 h of intermittent weight-bearing per day, thereby attenuating soleus muscle atrophy (Miyazaki et al. 2008). In this study, the HU+IWB+HS and HU+IWB groups were released from unloading for 1 h every second day because of a limitation in the experimental method; however, no significant changes in muscle weight or myofiber CSA, the number of TUNEL-positive nuclei, calpain autolysis, or ubiquitinated protein expression were observed between the HU and HU + IWB groups. Thus, the effect of intermittent weight-bearing on the reduction of muscle atrophy appears to have been minimal.

### Heat stress and heat shock proteins during muscle atrophy

Heat stress has attracted attention as an effective method to counter disuse muscle atrophy. Although the cellular mechanism responsible for heat stress–induced suppression of muscle atrophy remains unclear, HSPs play a key role in facilitating protein translation as molecular chaperones. In our study, HSP72 protein expression increased only in the plantaris muscle. Our data suggest that HSP72 plays an important role in regulating fast-type muscle atrophy, similar to slow-type soleus muscle atrophy (Naito et al. 2000; Goto et al. 2004; Selsby and Dodd 2005; Kojima et al. 2007; Takeda et al. 2009). Additionally, the difference between the soleus and plantaris muscles may have occurred because slow-type oxidative muscle has higher protein turnover than does other rat muscle types (Lewis et al. 1984; Garlick et al. 1989; van Wessel et al. 2010), and the HSP72 response occurs earlier in the soleus than in the plantaris muscle (Oishi et al. 2002). Thus, when we sacrificed the rats 2 days after the final heat treatment, soleus muscle HSP72 expression returned to a resting level, and no significant change was observed.

### Heat stress and the apoptotic system during muscle atrophy

Many studies have demonstrated that skeletal muscle atrophy is associated with increased apoptosis (Allen et al. 1996; Du et al. 2004; Powers et al. 2007). Consistent with previous studies that used the hindlimb unloading model, the number of TUNEL-positive nuclei increased significantly with disuse (Leeuwenburgh et al. 2005; Dupont-Versteegden et al. 2006). This result reflects an increase in the number of apoptotic nuclei associated with myofibers; therefore, the decrease in skeletal muscle weight and myofiber CSA occurred due to disuse muscle atrophy. Interestingly, we showed that the number of TUNEL-positive nuclei was attenuated by heat stress in atrophied rat soleus and plantaris muscles, indicating that heat stress contributes to suppress DNA fragmentation in atrophied rat skeletal muscle. The underlying mechanisms remain unclear, but HSPs play a role in heat stress–induced suppression of cellular apoptosis (Li et al. 2000; Beere and Green 2001; Garrido et al. 2006; Lanneau et al. 2008). Li et al. (Li et al. 2000) reported that HSP70 inhibited apoptosis downstream of cytochrome *c* release and upstream of caspase-3 activation in vitro (U937/HSP70 cells). Thus, our results suggest that heat stress is useful to attenuate the loss of muscle mass and size induced by disuse in vivo.

### Heat stress and proteolytic systems during muscle atrophy

One of our main finding was that heat stress–induced suppression of muscle atrophy was associated with lower levels of proteolytic markers such as calpain autolysis and subsequent protein ubiquitination in the soleus muscle of the heat stress-treated group. Ubiquitination of soleus myofibrillar protein increased specifically following short-duration hindlimb unloading (4 or 8 days), and muscle weight therefore decreased significantly (Vermaelen et al. 2005). Here, we found that ubiquitinated protein content increased in the soleus muscle, but that increase was attenuated in the heat-treated group. Thus, our results indicate that repeated heat stress was effective in reducing myofibrillar protein ubiquitination and attenuating atrophy of the soleus muscle.

Although muscle protein degradation during disuse is mainly due to activity of the ubiquitin–proteasome system (Furuno et al. 1990; Tiao et al. 1994), calpain is a key contributor to breakdown of myofibrillar protein by the proteasome. Calpains are Ca^2+^-dependent cysteine proteases that are activated during skeletal muscle atrophy (Powers et al. 2007). Indeed, calpains have no direct role in the degradation of contractile proteins such as actin and myosin, but they release myofibrillar proteins by cleaving cytoskeletal proteins such as desmin and *α*-actinin (Kandarian and Stevenson 2002). In our study, the reduction in desmin protein expression in atrophied soleus muscle was attenuated by heat treatment, possibly because repeated heat stress suppresses calpain activation and subsequent protein ubiquitination. The precise mechanism by which repeated heat stress attenuates calpain autolysis remains unclear but it may suppress calcium leakage from the sarcoplasmic reticulum induced by oxidative stress (Kondo and Itokawa 1994; Powers et al. 2007). An increase in intracellular calcium level activates the calpain system; therefore, repeated heat stress may suppress the calcium leak and subsequent calpain activation. Additionally, repeated heat stress may activate calpastatin, an endogenous calpain inhibitor; however, further studies are required to clarify the mechanism by which heat stress attenuates calpain activation.

As expected, we found that protein ubiquitination and calpain autolysis increased in the soleus muscle particulate fraction, whereas ubiquitination and calpain autolysis remained unchanged in the plantaris muscle. These differences could be associated with a less-atrophied muscle condition and the observation that fast-type muscles are less sensitive to disuse are slow-type and postural muscles (Thomason and Booth 1990; Kasper and Xun 1996). Our results are consistent with a previous report indicating that activation of calpain during hindlimb unweighting was elevated earlier in the slow-type soleus muscle than in the fast-type gastrocnemius muscle (Enns and Belcastro 2006); therefore, protein ubiquitination remained unchanged in the plantaris muscle.

We also detected no change in the lysosomal protease cathepsin L in either muscle of the experimental groups. The lysosome system does play a critical role in myofibrillar protein degradation during disuse (Taillandier et al. 1996); therefore, unloading and heat stress did not affect cathepsin L expression in our study.

### Conclusions and perspectives

In conclusion, our data demonstrate that (1) heat stress–induced suppression of apoptosis was responsible for attenuating soleus and plantaris muscle atrophy; and (2) repeated heat stress attenuated activation of proteolysis in slow-type soleus muscle but not in fast-type plantaris muscle. Our data suggest that heat stress attenuates disuse-induced muscle atrophy not only by upregulating HSP72, as shown in previous studies, but also by modulating apoptotic and/or proteolytic systems in slow- and fast-type muscles. The differences between the soleus and plantaris muscles could be related to higher protein turnover in slow-type muscle and less sensitivity to disuse in fast-type muscle. However, we could not perform the heat treatment during hindlimb unloading because of a limitation in the experimental method. Therefore, future studies are required to determine whether the effects were due to the combination of repeated heat stress and weight-bearing or due to heat stress alone. Further understanding of the mechanism is required to develop an effective heat treatment for disuse-induced muscle atrophy.

## Conflict of Interest

None declared.
